# Synergistic Effects of Controlled-Released BMP-2 and VEGF from nHAC/PLGAs Scaffold on Osteogenesis

**DOI:** 10.1155/2018/3516463

**Published:** 2018-09-24

**Authors:** Ting Wang, Shu Guo, Hua Zhang

**Affiliations:** Department of Plastic Surgery, The First Hospital of China Medical University, Shenyang 110001, China

## Abstract

Tissue engineering bones take great advantages in massive bone defect repairing; under the induction of growth factors, seed cells differentiate into osteoblasts, and the scaffold materials gradually degrade and are replaced with neogenetic bones, which simulates the actual pathophysiological process of bone regeneration. However, mechanism research is required and further developed to instruct elements selection and optimization. In the present study, we prepared vascular endothelial growth factor/bone morphogenetic protein-2- nanohydroxyapatite/collagen (VEGF/ BMP-2- nHAC/ PLGAs) scaffolds and inoculated mouse MC3T3-E1 preosteoblasts to detect osteogenic indexes and activation of related signaling pathways. The hypothesis is to create a three-dimensional environment that simulates bone defect repairing, and p38 mitogen-activated kinase (p38) inhibitor was applied and osterix shRNA was transferred into mouse MC3T3-E1 preosteoblasts to further investigate the molecular mechanism of crosstalk between BMP-2 and VEGF. Our results demonstrated the following: (1) BMP-2 and VEGF were sustainably released from PLGAs microspheres. (2) nHAC/PLGAs scaffold occupied a three-dimensional porous structure and has excellent physical properties. (3) MC3T3-E1 cells proliferated and differentiated well in the scaffold. (4) Osteogenic differentiation related factors expression of VEGF/BMP-2 loaded scaffold was obviously higher than that of other groups; p38 inhibitor SB203580 decreased the nucleus/cytoplasm ratio of osterix expression. To conclude, the active artificial bone we prepared could provide a favorable growth space for MC3T3-E1 cells, and osteogenesis and maturation reinforced by simultaneous VEGF and BMP-2 treatment may be mainly through the activation of the p38 MAPK pathway to promote nuclear translocation of osterix protein.

## 1. Introduction

Bone defect repair is a complex process involving several cell types as osteoblasts, endothelial cells, and so on, besides various growth factors [1]. Delivery of cells and growth factors towards the defect and preservation of their activity has already been proven to be the crux of the resolution [2, 3]. Tissue engineering has great advantages, cultivating seed cells in a natural or synthetic, biocompatible and degradable scaffold, then constructing active artificial bones under the induction of growth factors. After being implanted into bone defect cavity, new bones gradually generate with the degradation of scaffold material, which can finally meet reconstructive objectives [4, 5].

Configuration and crystal size of nanohydroxyapatite (nHA) are similar to the natural bone that holds the advantage of nice biocompatibility, osteogenic activity, and osteoinduction. But slow degradation that impairs new bone formation restricts its application. The degradation rate, biocompatibility, and plasticity of collagen are all excellent, while its mechanical strength is poor [6, 7]. Combination of nHA and collagen is expected to break through the physic-chemical defects of the two materials and meet the requirements of ideal artificial bone scaffold [8]. Among the few BMP products approved by Food and Drug Administration (FDA) for clinical use, recombinant human bone morphogenetic protein-2 (rhBMP-2) possesses potent osteogenic ability, which can induce bone and cartilage formation both in vivo and in vitro [9, 10]. However, well-orchestrated regulation of various cytokines can finally achieve satisfactory reconstructive outcomes [1]. Vascular endothelial growth factor (VEGF) can not only promote angiogenesis, but also strengthen differentiation and maturation of osteocytes [11]. It is reported that BMP-2 interacts with VEGF during bone regeneration [12, 13]. Therefore, simultaneous application of BMP-2 and VEGF is expected to enhance the repair effect of a single one. However, their short half-life and poor stability will lead to complications such as ectopic osteogenesis, hematoma, and hemangioma in case of excessive release [14, 15]. Poly(lactide-co-glycolide) (PLGA) has been used as a suture material for surgical operations. Because of low immunogenicity, low toxicity, controllable degradability, high encapsulation rate, and sustainable drug release, PLGAs have been widely applied as drug carrier in scientific researches and clinical treatment [16]. However, foreign body reactions and aseptic inflammation caused by its acid degradation products will inhibit bone formation [17]. The combination of nHA may improve this deficiency by providing hydroxyl groups to neutralize the acidic environment [18]. In the present study, we combined BMP-2/ VEGF loaded PLGA microspheres with nHA and collagen, hoping to prepare bone tissue engineering scaffolds with excellent physicochemical and biological properties.

MC3T3-E1 cells, a mouse preosteoblast cell line that has been widely used as a good model for studying osteogenic differentiation in vitro, especially under the actin of ECM signaling pathways [19], have been inoculated into BMP-2/ VEGF sustainably released nHA/ PLGA microspheres/collagen scaffold (abbreviated as nHAC/ PLGAs), then we carried out in vitro experiments to evaluate the osteogenic effects and mechanisms of BMP-2 and VEGF crosstalk in 3D environment.

## 2. Materials and Methods

### 2.1. Preparation of Porous Three-Dimensional nHAC/PLGAs Scaffold

PLGA microspheres were fabricated by a modified water- oil- water (w/o/w) double emulsion solvent evaporation method: The primary organic phase was prepared by dissolving PLGA (Sigma) in dichloromethane (100 g/L). VEGF or BMP-2 (PeproTech) was dissolved in bovine serum albumin (BSA, Sigma) solution (w_BSA_: w_PLGA_ =0.6) to form inner aqueous phase. The primary emulsion phase was prepared by mixing primary organic phase and inner aqueous phase (V_organic_: V_aqueous_=0.2) using a probe type sonicator (Kinematica) for 60 s in an ice bath to form double emulsion, then aqueous PVA solution (40 g/L) was added (V_organic_: V_PVA_=0.5) and mixed again to form double emulsion phase using a homogenizer (Heidolph) for 180s at 5000 rev/min in an ice bath. The double emulsion was poured into diluted aqueous PVA solution (4 g/L, V_emulsion_: V_PVA_=0.05) and mixed thoroughly using a magnetic stirrer at 800 rev/min for 3 h at room temperature to evaporate organic solvent. The solidified microspheres were collected by centrifugation (5000g, 10 min), washed with deionized water three times, prefrozen at −20°C overnight, and then lyophilized at −25°C and 10 Pa for 48 h.

Tails of rats were soaked in 75% ethanol for 10 min and then washed with 0.9% sodium chloride solution three times. After skin was peeled, tendons were extracted, soaked in 75% ethanol for 15 min, and then washed with deionized water repeatedly. The tendons were trimmed using scissors into lmm^3^ tissue mass, soaked in 0.05M Tris / HCL solution (pH 7.5), and then stored at 4°C overnight. Tris / HCL was replaced with 0.05 M acetic acid solution (pH 4.76, 2 L acetic acid for 5 g tendon) and placed at 4°C for 4 days. The solution was centrifuged (4°C, 320 g, 10 min); the obtained supernatant was filtered through a 50 um mesh to acquire crude collagen solution. 0.14mol / L NaOH solution was added to the above solution (V_NaOH_:V_collagen_=1:6) and centrifuged (4°C, 12000 g, 15 min). The supernatant was discarded, and the flocculent precipitate was collected and redissolved in 200 ml 0.05M acetic acid solution. The above solution was embedded into dialysis bag and immersed in deionized water for one week; the eluate is changed every 12 h. Finally, the purified collagen solution was prefrozen at −20°C overnight and then lyophilized at −25°C and 10 Pa for 48 h.

Rat tail collagen was dissolved in deionized water to form collagen solution (20mg/ml). nHAP powder (Nanjing Emperor Nano Material Co., Ltd) was homodispersed in collagen solution (w_collagen_: w_nHA_= 7:3) using magnetic stirrer. BMP-2 or VEGF loaded and unloaded PLGA microspheres were then homodispersed in the above solution (10mg/ml) using magnetic stirrer. The above compound was added to the pores (8 mm diameter and 8 mm high) of polytetrafluoroethylene mould (Suzhou YKJ Mould Technology Co., Ltd), prefrozen at −20°C overnight, and then lyophilized at −25°C and 10 Pa for 48 h to acquire scaffold.

### 2.2. Characterization of BMP-2/VEGF Loaded nHAC/ PLGAs Scaffold

The surface and internal morphology of the scaffold were observed and photographed using scanning electron microscopy (SEM; Hitachi) after the samples were broken and sputter-coated with gold. Then SEM images were analyzed by ImageJ software to measure pore size and microsphere particle diameter. The scaffold was immersed into hexane (primary volume was V1) to be impregnated, the total volume of hexane and scaffold was V2; the residual hexane volume was V3 after removing hexane-impregnated scaffolds. The porosity of the scaffold was calculated as (V1-V3)/(V2-V3) × 100. Water absorption rate (W) of the scaffolds was measured according to the following equation (m1 indicates dry weight of samples; m2 indicates wet weight of samples saturated with water in air): W=(m2-m1)/m1 × 100%. The compressive strength and elastic modulus of the scaffold were acquired by calculating the stress-strain curve measured using universal mechanical properties testing instrument (ZWICKZ005).

### 2.3. Releasing Characteristics of BMP-2/VEGF from Microspheres

20 mg growth factor loaded microspheres were immersed in 2 ml PBS and then oscillated in 37°C constant temperature water bath for 28 days. 1 ml supernatant was aspirated and replaced with fresh phosphate buffer saline (PBS, pH 7.4, Hyclone) every 24 h. The amount of released BMP-2 or VEGF in the supernatant was detected, respectively, using ELISA assay kit (R&D) according to the instructions. After being washed, diluted standards or samples were added into each well, followed by being incubated with diluted detected antibody at room temperature for 2 hours. Then wells were washed again 6 times and filled with diluted Streptavidin-HRP to incubate at room temperature for 45 minutes, followed by being incubated with substrate solution away from light for 30 minutes at room temperature. After adding stop solution, the optical density of wells can be determined using microplate reader under double wavelength of 450 and 570/ 630 nm within 30 minutes. Concentration of samples can be calculated according to a formula derived from concentration and optical density of standards, then accumulative release curves were plotted to describe the release behavior of BMP-2/VEGF.

### 2.4. Biocompatibility of nHAC/ PLGAs Scaffold

MC3T3-E1 cells (purchased from National Infrastructure of cell line resource) were inoculated into the scaffold and incubated with common culture medium (Dulbecco's Modified Eagle's Medium-high glucose plus 10% fetal bovine serum) for 1, 3, and 5 days, then samples were fixed with 2.5% glutaraldehyde overnight and then dehydrated with alcohol (30%, 50%, 70%, 80%, 90%, 95%, 100%, and 100%, each for 10 min) to be observed under SEM.

### 2.5. In Vitro Osteogenic Activity of MC3T3-E1 Cells under Different Conditions

MC3T3-E1 cells were inoculated into the scaffold at a density of 1 × 10^8^/ml and cultured in common medium with or without BMP-2 (1 *μ*g primary loaded in the scaffold) or VEGF (400ng primary loaded in the scaffold) under standard culture conditions for subsequent detection. The experimental groups were as in Table 1.

#### 2.5.1. Determination of Proliferative Activity

At the 3rd day, adherent cells were separated from scaffold using trypsin and resuspended with common culture medium. Cell suspension and 1/10 volume of cell counting kit- 8 (CCK-8) solution (Beyotime) were added to a 96-well plate and incubated at 37°C for 2 h. Then absorbance was detected using microplate reader under 450 nm wavelength to reveal proliferation of MC3T3-E1 cells.

#### 2.5.2. Determination of ALP Activity

At 7th, 14^th^, and 21th days, adherent cells were separated from scaffold by 0.05% Triton X-100 and ultrasonic treatment (150W, 250s) in ice bath. Then the supernatant was collected after centrifugation (4°C, 12000 g, 15 min), 20 *μ*l samples were used to measure total protein concentration by bicinchoninic acid (BCA) protein assay kit, then we can calculate the protein mass. 50 *μ*l samples and 50 *μ*l para-nitrophenyl phosphate (pNPP, Beyotime, dissolved in diethanolamine (DEA) buffer, pH 9.8) were added to 96-well plates, followed by incubation at 37°C for 30 min. After adding 100 *μ*l termination reaction solution, optical density was detected using microplate reader under 405 nm wavelength. DEA enzyme activity unit was calculated according to formula derived from concentration and optical density of standards per unit time. Alkaline phosphatase (ALP) activity was expressed by the DEA activity unit divided by protein mass.

#### 2.5.3. Determination of Mineralized Nodules

At the 21th day, adherent cells were separated from scaffold using trypsin, then inoculated, and cultured in culture plate for 21 days. Cells were fixed and then stained using 0.2% alizarin red solution (Solarbio, pH 8.3). Formation of mineralized nodules was observed under microscope (Olympus).

#### 2.5.4. Expression of Osteogenic Differentiation Related Factors at mRNA Level

At the 7th, 14th, and 21th days, cell-scaffold complex was trimmed, then immersed in RNAiso Plus (TaKaRa), and triturated evenly. The supernatant was collected after being centrifuged (4°C, 12000 g, 15 min), mixed with chloroform (V_chloroform_:V_RNAiso  Plus_= 1:5), then stood for 5 min at room temperature. The upper water phase was separated after being centrifuged (4°C, 12000 g, 15 min), mixed with equal volume of isopropanol (V_isopropanol_:V_RNAiso  Plus_= 1:5), then stood for 10 min at room temperature. The precipitant was collected after being centrifuged (4°C, 12000 g, 15 min) and washed twice with 75% ethanol. After dehydration, the precipitant was dissolved in diethyl pyrocarbonate (DEPC) water. The concentration of mRNA was measured by adding 1 *μ*l sample to spectrophotometer. The reverse transcriptase reaction was performed, and the obtained cDNA was used for the subsequent real time PCR reaction according to the manufacturer's instructions. Sequences of primers used in this study were listed as follows:

  mouse GAPDH (Forward: 5′- CTTTGTCAAGCTCATTTCCTGG - 3′;
    Reverse: 5′- TCTTGCTCAGTGTCCTTGC - 3′)
  mouse Dlx-5 (Forward: 5′- AGCTACCTGGAGAACTCGG - 3′;
    Reverse: 5′- CCCAAAACTGAGCAAGAGAAAG - 3′)
  mouse osterix (Forward: 5′- CCTCTCCCTTCTCCCTCTC -3′;
    Reverse: 5′- CTGGAGCCATAGTGAGCTTC -3′)
  mouse col1 (Forward: 5′- CATAAAGGGTCATCGTGGCT - 3′;
    Reverse: 5′- TTGAGTCCGTCTTTGCCAG - 3′)

#### 2.5.5. Expression of Osteogenic Differentiation Related Factors and Signaling Pathway Molecules at Protein Level

At the 7th, 14th, and 21th days, cell-scaffold complex was trimmed, then immersed in lysate (RIPA lysis buffer with 1 mM PMSF, Beyotime), and ultrasonically homogenized on ice to extract total protein. Concentration of protein samples was measured using BCA protein assay kit. After being mixed with loading buffer, protein samples were denatured in 95°C water bath for 10 min. Protein bands (30*μ*g per lane) were separated by electrophoresis and transferred on PVDF membranes to be incubated with the following primary antibodies: *β*-actin (Proteintech), Runt-related transcription factor 2 (Runx-2), Distal-less homeobox 5 (Dlx-5), osterix (Abcam), collagen I (col1, Merck Millipore), Smad1/5/9, phosphorylated-Smad1/5/9 (pSmad1/5/9), serine/threonine kinase (Akt), phosphorylated-Akt (pAkt), p38 mitogen-activated kinase (p38), and phosphorylated-p38 (p-p38, Cell Signaling) at 4°C overnight and corresponding second antibodies under room temperature for 2 hours, developed using ECL gel imaging system. The grey level of protein bands was measured using ImageJ software; relative expression of the above indexes was determined by their grey levels divided by that of *β*-actin.

#### 2.5.6. Osteogenic Differentiation after Inhibition of Related Signaling Pathways or Osterix Knockdown

p38 inhibitor SB203580 and Akt inhibitor LY294002 (20*μ*M, Cell Signaling) were, respectively, added to a common medium. At the 21th day, the expression of col1 protein and mineralized nodules were detected. Expression of Dlx-5 and osterix protein was detected at the 21th day of culture in addition to SB203580. osterix shRNA (OriGene) were transfected into MC3T3-E1 cells by lentivirus. Cells were cultured in a 6-well plate until reaching 50% fusion; the common medium was discarded and replaced with a transfection medium (pure DMEM without FBS mixed with osterix shRNA loaded lentivirus). After 12 h, the transfection medium was discarded and replaced with a common medium. Cells were then inoculated into scaffold; the expression of col1 protein and mineralized nodules were detected at the 21th day.

### 2.6. Statistical Analysis

Data were analyzed using SPSS package 20.0. Comparisons among the groups were analyzed with independent sample* t*-test or one-way ANOVA. P <0.05 was considered statistically significant; P <0.01 was considered obviously statistically significant.

## 3. Results

### 3.1. Physical Properties of nHAC/PLGAs Scaffold

**Figure 1:**
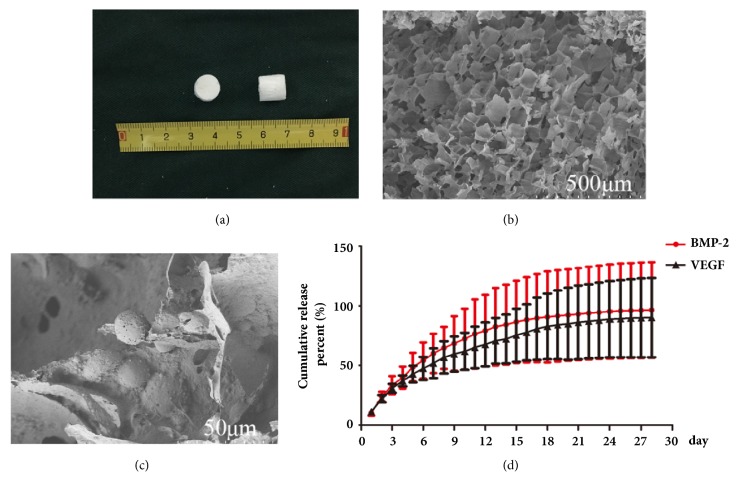
(a) General appearance and surface topography under SEM, (b) ×100, (c) ×900 of nHAC/PLGAs scaffold, and (d) releasing characteristics of BMP-2 and VEGF from PLGA microspheres.

nHAC/PLGAs scaffold displayed a cylindrical shape with a diameter of 0.8cm and height of 0.8cm, being milky white and rough and having a foamy surface (Figure 1(a)). Under SEM, nHAC/PLGAs scaffolds showed a three-dimensional porous structure with pore size ranging from tens of um to nearly 300 um. The pore wall displayed rough appearance with scattered nHA particles. PLGA microspheres with a mean diameter of 9.95um were uniformly dispersed, embedded in or attached to the pore wall (Figures 1(b) and 1(c)). The porosity of the scaffolds was 79.46%, and the water absorption rate was high up to 561.51%. The compressive strength of the scaffolds was 2.42 MPa, and the elastic modulus was 13.65 MPa (Table 2).

### 3.2. Releasing Characteristics of BMP-2/VEGF

Burst release was observed during the first 7 days; the released amount of BMP-2 and VEGF was 60.20% and 51.98%, respectively. A steady release was observed between the 8th and 17th days; the released amount of BMP-2 and VEGF was 29.53% and 28.82%, respectively. Only 6.85% of BMP-2 and 9.37% of VEGF were released during the remaining 11 days (Figure 1(d)).

### 3.3. Biocompatibility of nHAC/PLGAs Scaffold

**Figure 2:**
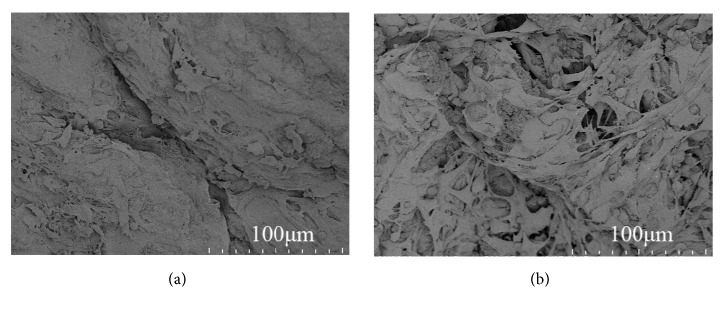
MC3T3-E1 cells growing in nHAC/PLGAs scaffold for 1 day (a) and 5 days (b).

At the first day under SEM observation, a small number of MC3T3-E1 cells were observed; they displayed ovary or irregular morphology and loosely stuck on the scaffold surface (Figure 2(a)). At the 5th day, the quantity of MC3T3-E1 cells increased; they stretched and displayed polygonal or elongated morphology and tightly stuck on the scaffold surface (Figure 2(b)).

### 3.4. In Vitro Osteogenic Activity and Mechanism Investigation of MC3T3-E1 Cells in nHAC/PLGAs Scaffold

#### 3.4.1. Comparison of Proliferative Activity

**Figure 3:**
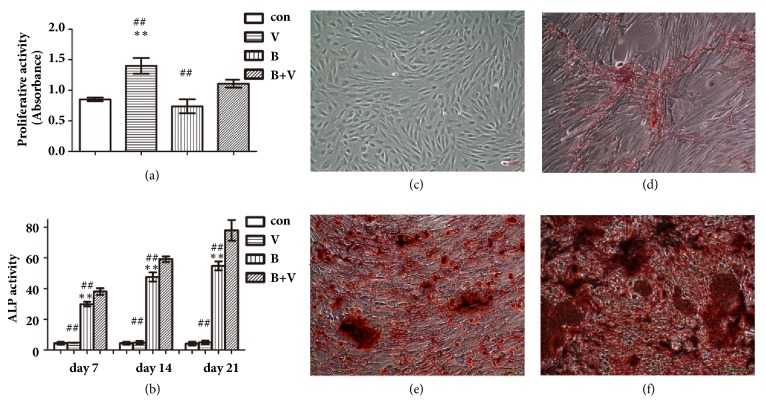
Proliferative activity (a) and ALP activity (b) of MC3T3-E1 cells in nHAC/PLGAs scaffold (con: control group; V: VEGF group; B: BMP-2 group; B+V: BMP-2/VEGF group) (*∗∗*P < 0.01 compared with control group; ^##^P < 0.01 compared with BMP-2/VEGF group). Calcium nodules detection of MC3T3-E1 cells in nHAC/PLGAs scaffold ((c) control group; (d) VEGF group; (e) BMP-2 group; (f) BMP-2/VEGF group).

Compared with the control group, the proliferative activity of VEGF group was higher (P<0.01), but that of BMP-2 group was similar (P>0.05). The proliferative activity of BMP-2/VEGF group was higher (P<0.01) than that of BMP-2 but lower (P<0.01) than that of the VEGF group (Table 3, Figure 3(a)).

#### 3.4.2. Comparison of ALP Activity

At the 7th day, compared with the control group, the ALP activity of BMP-2 group was significantly higher (P<0.01), but that of VEGF group was similar (P>0.05). ALP activity of BMP-2/VEGF group was significantly higher (P<0.01) than that of the BMP-2 and VEGF groups. At the 14th and 21th days, the ALP activity of each group displayed similar trends to that at the 7th day (Table 3, Figure 3(b)).

#### 3.4.3. Formation of Mineralized Nodules

At the 21th day, cells in the control group displayed elongated morphology and were randomly arranged; they almost cannot be stained by alizarin red. Cells in the other three groups were arranged in a turbo form, with orange colored calcium nodules deposited in the extracellular matrix. The calcium nodules in the VEGF group were small in number and volume; they were scattered and lightly colored. The number and volume of calcium nodules in the BMP-2 group were bigger than those of the VEGF group, with several calcium nodules clustered together. Calcium nodules were filled with culture plate of BMP-2/VEGF group, with most calcium nodules clustered together and deeply colored (Figures 3(c)–3(f)).

#### 3.4.4. Expression of Col1 mRNA and Protein

**Figure 4:**
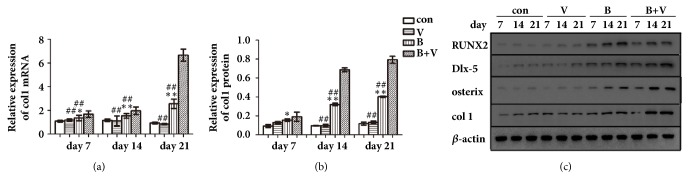
Relative expression of col1 mRNA (a), col1 protein (b), and western blot images of RUNX2, Dlx-5, osterix, col1, and *β*- actin protein (c) of MC3T3-E1 cells in nHAC/PLGAs scaffold (*∗*P < 0.05 and *∗∗*P < 0.01 compared with control group; ^#^P < 0.05 and ^##^P < 0.01 compared with BMP-2/VEGF group; con: control group; V: VEGF group; B: BMP-2 group; B+V: BMP-2/VEGF group).

At the 7th day, compared with the control group, the relative expression of col1 mRNA and protein in BMP-2 group was higher (P<0.05), but that of VEGF group was similar (P>0.05). Col1 mRNA (P<0.01) and protein (P>0.05) expression of BMP-2/VEGF group were significantly or slightly higher than those of the BMP-2 and VEGF groups. At the 14th and 21th days, relative expression of col1 mRNA and protein of each group displayed similar trends to that at the 7th day, except for their expression in BMP-2/VEGF group which was significantly higher than that of BMP-2 and VEGF groups (P<0.01) (Table 3, Figure 4).

#### 3.4.5. Expression of Osteogenic Related Factors at mRNA and Protein Level

**Figure 5:**
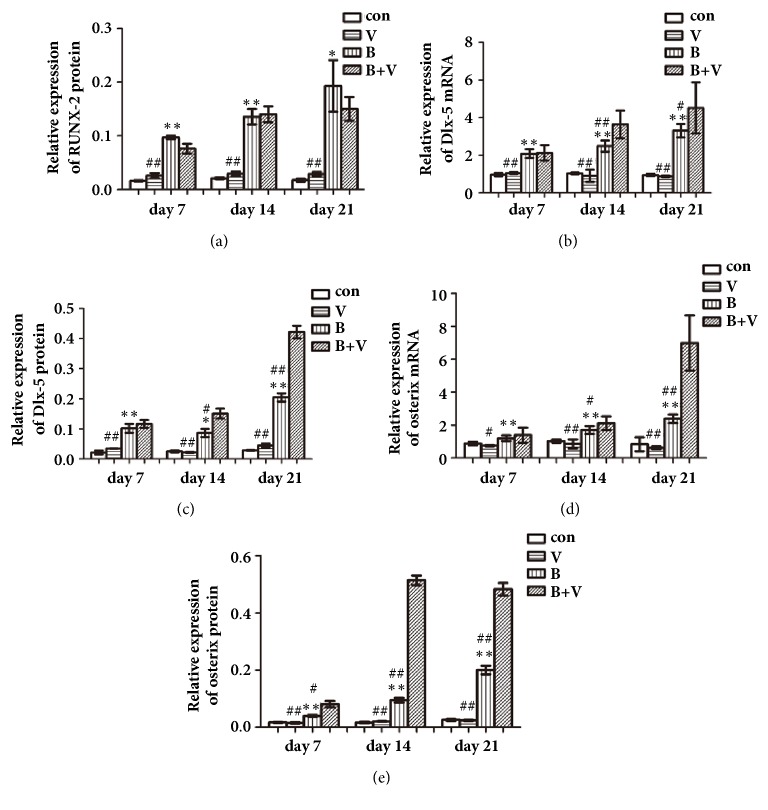
Relative expression of RUNX2 protein (a), Dlx-5 mRNA (b), Dlx-5 protein (c), osterix mRNA (d), and osterix protein (e) of MC3T3-E1 cells in nHAC/PLGAs scaffold (*∗*P < 0.05 and *∗∗*P < 0.01 compared with control group; ^#^P < 0.05 and ^##^P < 0.01 compared with BMP-2/VEGF group; con: control group; V: VEGF group; B: BMP-2 group; B+V: BMP-2/VEGF group).

At the 7th day, compared with the control group, the relative expression of RUNX-2 protein in BMP-2 group was significantly higher (P<0.01), but that of the VEGF group was similar (P>0.05). RUNX-2 protein expression of BMP-2/VEGF group was similar (P>0.05) to that of BMP-2 group and significantly higher (P<0.01) than that of VEGF group. At the 14th and 21th days, the relative expression of RUNX-2 protein of each group displayed similar trends to that at the 7th day (Table 3, Figures 4(c) and 5(a)).

At the 7th day, compared with the control group, the relative expression of Dlx-5 mRNA and protein in BMP-2 group was significantly higher (P<0.01), but that of VEGF group was similar (P>0.05). Dlx-5 mRNA and protein expression of BMP-2/VEGF group were similar (P>0.05) to that of BMP-2 group and significantly higher (P<0.01) than that of VEGF group. At 14th and 21th days, relative expression of Dlx-5 mRNA and protein of each group displayed similar trends to that of the 7th day, except for their expression of BMP-2/VEGF group that was higher (P<0.05 or P<0.01) than that of BMP-2 group (Table 3, Figures 4(c), 5(b), and 5(c)).

At 7th day, compared with control group, relative expression of osterix mRNA and protein in BMP-2 group was significantly higher (P<0.01), but that of VEGF group was similar (P>0.05). Osterix mRNA and protein expression of BMP-2/VEGF group were higher than that of VEGF group and slightly higher than that of BMP-2. At 14th and 21th days, relative expression of osterix mRNA and protein of each group displayed similar trends to that at the 7th day, except for their expression in BMP-2/VEGF group that was significantly higher than that of BMP-2 and VEGF group (P<0.01) (Table 3, Figures 4(c), 5(d), and 5(e)).

#### 3.4.6. Expression of Osteogenic Related Signaling Pathway

**Figure 6:**
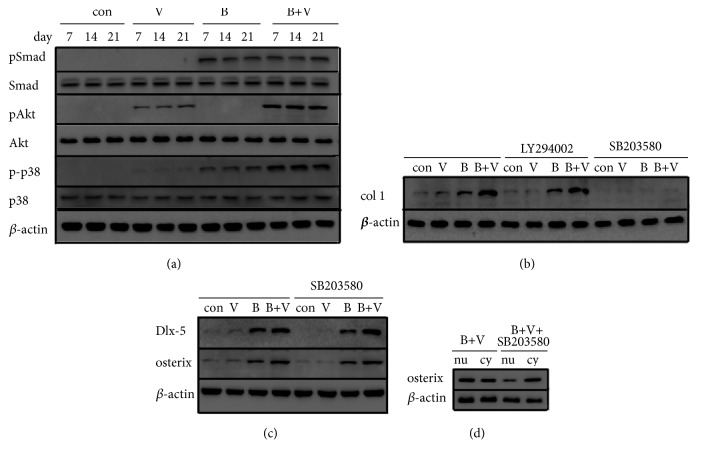
(a) Expression of osteogenic related signaling pathway proteins of MC3T3-E1 cells in nHAC/PLGAs scaffold. (b) Expression of col1 protein of MC3T3-E1 cells treated with LY294002 and SB203580. (c) Expression of Dlx-5 and osterix protein of MC3T3-E1 cells treated with SB203580. (d) Expression of osterix protein in nucleus and cytoplasm of MC3T3-E1 cells in BMP-2/VEGF group (con: control group; V: VEGF group; B: BMP-2 group; B+V: BMP-2/VEGF group; nu: nucleus, cy: cytoplasm).

pSmad expression was almost not detected in control group and VEGF group, while it was obviously detected in BMP-2 and BMP-2/VEGF group, but there was no significant difference between them. pAkt expression was almost not detected in control group and BMP-2 group, while it was obviously detected in VEGF and BMP-2/VEGF group, and its expression in BMP-2/VEGF group was significantly higher than that of VEGF group. p-p38 expression was detected in VEGF, BMP-2,and BMP-2/VEGF group; its expression in BMP-2 group was significantly higher than that in VEGF group, and its expression in BMP-2/VEGF group was significantly higher than that in VEGF and BMP-2 group. There was a little difference among the expressions of signal pathway proteins at different stages of osteogenesis; thus, the interaction between VEGF and BMP-2 enhanced the activation of PI3K/Akt and p38 MAPK pathways (Figure 6(a)).

#### 3.4.7. Mechanism Investigation of BMP-2 and VEGF Crosstalk on Osteogenesis

**Figure 7:**
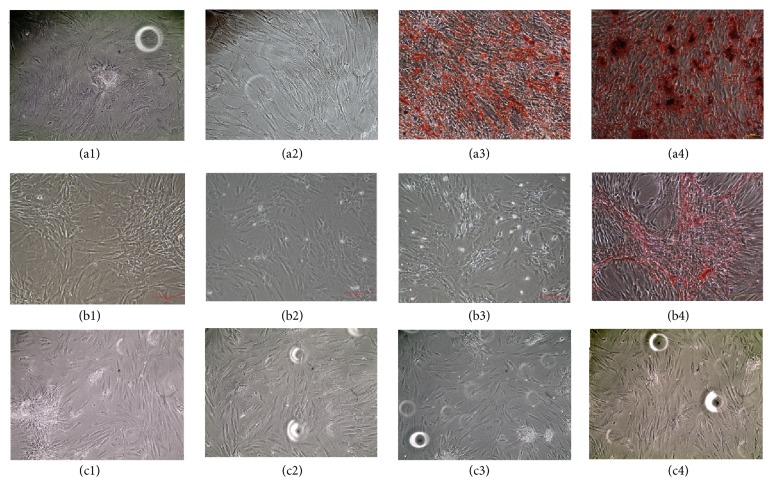
Calcium nodules detection of MC3T3-E1 cells treated with (a) LY294002, (b) SB203580, and (c) osterix shRNA in nHAC/PLGAs scaffold (1: control group; 2: VEGF group; 3: BMP-2 group; 4: BMP-2/VEGF group).

At the 21th day, under treatment with LY294002, no calcium nodules were detected in control group and VEGF group, only a few lightly-colored calcium nodules scattered in BMP-2 and BMP-2/VEGF group, and col1 protein expression in all groups changed a little (Figures 6(b) and 7(a)). Under treatment with SB203580, almost no calcium nodules were observed, and col1 protein expression significantly decreased in all groups, and expression of Dlx-5 and osterix protein changed little, while the nucleus/cytoplasm ratio of osterix expression decreased in BMP-2/VEGF group (Figures 6(b), 6(c), 6(d), and 7(b)).

**Figure 8:**
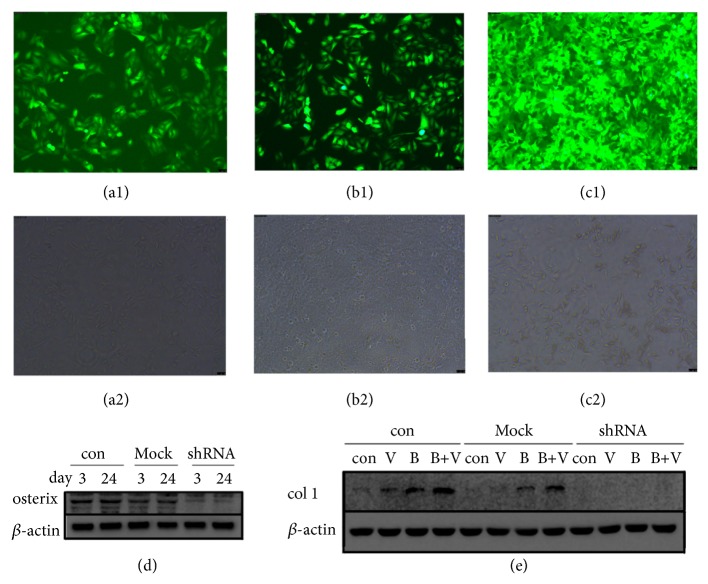
Expression of GFP protein in MC3T3-E1 cells treated with osterix shRNA unloaded ((a) 3rd day after transfection) or loaded ((b) 3rd day and (c) 24th day after transfection) lentivirus (1. Images in fluorescent state; 2. Images in ordinary state). (d) Osterix protein expression of MC3T3-E1 cells treated with osterix shRNA loaded or unloaded lentivirus at the 3rd day and 24th day after transfection. (e) Expression of col1 protein in MC3T3-E1 cells treated with osterix shRNA loaded or unloaded lentivirus at 24th day after transfection (con: control, Mock: unloaded lentivirus; shRNA: loaded lentivirus).

Osterix knockdown by lentivirus carrying osterix shRNA can be stable in our experimental period (Figures 8(a)–8(d)). At the 21th day, almost no calcium nodules and col1 protein expression of osterix knockdown MC3T3-E1 cells were detected in all groups (Figures 7(c) and 8(e)).

## 4. Discussion

Collagen, which constitutes a skeleton of extracellular matrix and participates in cellular vital activities, possesses strong toughness, high tensile strength, and low immunogenicity; thus, it has been considered as an ideal material in tissue engineering. As the parent phase of the scaffold, collagen interweaves into a three-dimensional porous network structure [20], which is beneficial to the attachment of nHA and PLGA microspheres and seed cells. However, poor bending strength and overquick degradation rate of collagen confine its further application [21, 22]. Addition of nHA provided ergonomic strength and potent osteoconduction and osteoinduction [23–25]. The combination of nHA and collagen can make up the mutual defects and has better mechanical and biological properties. Due to high surface activity, nHA can uniformly disperse in the collagen parent phase, and they form excellent interface interaction: the collagen has high affinity with Ca^2+^; thus, nHA is recruited and distributed along collagen fibers that act as crystal nucleus and mineralization template in the crystallization process of nHA. nHA provides free hydroxyl groups, which increase water absorption of the composite material and, thus, promote cell attachment, protein adsorption, and nutrition transfer [26]. More importantly, as a nucleating site, nHA causes mineral deposition and, thus, gives osteogenic potential to collagen-based scaffold material [27]. Given these, combination of nHA and collagen provided the material excellent physical properties to bear certain weight and provide space for seed cells.

The PLGA microspheres we prepared take advantage of concentrating loaded BMP-2/ VEGF inside the shell, which can achieve sustainable release by dissolution degradation. These PLGA microspheres and nHA dispersed in the collagen; the hydrophilic surrounding environment and polar reaction as well as hydrogen bond among these molecules further help maintain the stability of loaded growth factors [28].

Researchers have reported that 3D culture can provide cells with a similar microenvironment as that in vivo and avoid contact inhibition and spontaneous senescence [29]. In the present study, MC3T3-E1 cells can adhere and proliferate in the nHAC/PLGAs scaffold, which indicated that the composite material possessed favorable biocompatibility. Among the osteogenic indicators we detected, ALP activity and col1 expression indicate osteogenesis at early stage. The formation of calcium nodules confirms maturation of new newborn bone and differentiated osteoblasts [30]. In the present study, we found that released VEGF alone stimulated proliferation but had no effect on osteogenic activity of MC3T3-E1 cells, while BMP-2 had opposite effects; combination of BMP-2 and VEGF further promoted osteogenesis in 3D environment provided by the porous scaffold. Any deficiency in osteodifferentiating cells, VEGF or BMPs would fail to repair bone defect [31]; thus, they dominate among the factors participating in the process of bone reconstruction. In combination of previous studies, involvement of VEGF did not directly mediate osteogenesis, but enhanced maturation of osteodifferentiated cells in the premise BMP-2 existing [32, 33]. RUNX-2 and Dlx-5 are identified osteogenic transcription factors that were upregulated under the activation of BMP/ Smad dependent or nondependent pathways [34], followed by increasing expression of osterix, then accumulation of osteogenesis and maturation indicators [35, 36]. CHING-JU LI et al. [23] have reported that crosstalk between the VEGF and BMP-6 pathways enhanced osteoblastic differentiation of human adipose-derived stem cells (hADSCs). They found that combination of VEGF and BMP-6 enhanced col1 expression, which correlated with upregulated expression of osterix, Dlx5 but not RUNX-2 in hADSCs. Combined with previous researches that BMPs can induce osterix expression in RUNX-2 deficient cells [37], our present investigation showed that BMP-2 should enhance the expression of osterix in MC3T3-E1 cells through Dlx5 upregulation. Inspired by their experimental philosophy [23], we detected osteogenesis-related Smad, p38MAPK, and PI3K/Akt pathway proteins and found upregulated activation of p38MAPK and PI3K/Akt pathway, but no obvious change of Smad pathway. Furthermore, osteogenesis of MC3T3-E1 cells was greatly disturbed by p38 inhibitor SB203580, but not affected by Akt inhibitor LY294002. As researches report, inhibiting Akt1 activity or knockout Akt1 gene may increase MSCs mineralization and ALP activity in mice, which can be reversed by importing Akt1via lentivirus; besides, Akt2 have opposite effect to Akt1 in osteogenesis, and inhibiting Akt2 can repress osteoblast differentiation [38, 39]. Contrary to their findings that PI3K/Akt pathway was attenuated through BMP-6/ VEGF crosstalk, we may attribute to different targets of BMP-2 and BMP-6 on Akt proteins or different action intensity between them and finally exhibit opposite effects. Given the results in the present study, the interaction between VEGF and BMP-2 is mediated by p38 MAPK pathway. However, expression of Dlx-5 and osterix protein in all groups changed a little, which indicates that the osteogenic effects caused by the activation of p38 MAPK pathway were not directly mediated by increasing osteogenic transcription factors expression. Besides, osterix plays a key role in the interaction between VEGF and BMP-2 on osteogenesis, as we found a complete inhibition of osteogenesis in osterix knockdown MC3T3-E1 cells. Furthermore, SB203580 treatment decreased the nucleus/cytoplasm ratio of osterix expression in BMP-2/VEGF group. Recent reports suggested that osterix phosphorylated by p38 leads to its nuclear translocation, initiating transcript of the target osteogenic factors, thus promoting osteogenesis as well as bone formation [40, 41]. In combination with all the above, we speculate that phosphorylation of osterix under p38 MAPK pathway activation may be the key to enhance the osteogenic differentiation and maturation of MC3T3-E1 cells through the interaction of VEGF and BMP-2.

## 5. Conclusions

Our results show that BMP-2 and VEGF sustainably released nHAC/ PLGAs scaffold possessed excellent physical properties and biocompatibility to bear pressure from the surrounding tissues and provide space for seed cells to attach and grow. In the 3D microenvironment provided by the scaffold, VEGF alone significantly promoted MC3T3-E1 cells proliferation, BMP-2 alone significantly promoted MC3T3-E1 cells osteogenic differentiation in nHAC/ PLGAs scaffold, and osteoinductive effect of simultaneous VEGF and BMP-2 treatment further enhanced, which may be mainly through increasing nuclear translocation of osterix protein by activation of the p38 MAPK pathway.

## Figures and Tables

**Table 1:** 

	MC3T3-E1 cells	scaffold	BMP-2	VEGF
A	+	_	_	_
B	+	+	+	_
C	+	+	_	+
D	+	+	+	+

**Table 2:** Physical parameters of nHAC/PLGAs scaffold($\overset{-}{x}\pm s$, n=3).

Pore size (*μ*m)	Diameter of PLGAs (*μ*m)	Porosity (%)	Water absorption rate (%)	Elastic modulus (MPa)	Compressive strength (MPa)
176 ± 93	9.95 ± 1.93	79.46 ± 7.78	561.51 ± 19.59	0.72 ± 0.17	2.85 ± 0.49

**Table 3:** Proliferative activity (OD value) and ALP activity (DEA enzyme activity, *μ*mol*∙*min^−1^*∙*g^−1^), relative expression of col1, Dlx-5, and osterix mRNA, relative expression of RUNX2, Dlx-5, osterix, and col1 protein of MC3T3-E1 cells in nHAC/PLGAs scaffold.

	Time (days)	Control group	VEGF group	BMP-2 group	BMP-2/VEGF group
Proliferative activity	3	0.8507 ± 0.0317	1.3987 ± 0.1304*∗∗*##	0.7385 ± 0.1160##	1.1096 ± 0.0632

ALP activity	7	4.2738 ± 0.8482	4.6884 ± 0.0979##	29.8862 ± 1.4436*∗∗*##	38.0940 ± 2.1503
	14	4.3097 ± 0.8896	4.8018 ± 1.0833##	47.5750 ± 3.0548*∗∗*##	59.1847 ± 1.8423
	21	4.0477 ± 1.1751	4.9057 ± 1.1357##	54.8533 ± 2.9016*∗∗*##	78.0085 ± 6.8198

Relative expression of col 1 mRNA	7	1.0927 ± 0.0758	1.1907 ± 0.0939##	1.3614 ± 0.2367*∗*##	1.6776 ± 0.2768
	14	1.1592 ± 0.1075	1.1095 ± 0.4211##	1.5429 ± 0.1926*∗∗*##	1.9635 ± 0.3183
	21	0.9377 ± 0.0719	0.8569 ± 0.0447##	2.5563 ± 0.3960*∗∗*##	6.6649 ± 0.5074

Relative expression of col 1 protein	7	0.0935 ± 0.0269	0.1288 ± 0.0201	0.1551 ± 0.0235*∗*	0.1908 ± 0.0840
	14	0.0968 ± 0.0043	0.0963 ± 0.0277##	0.3205 ± 0.0238*∗∗*##	0.6859 ± 0.0372
	21	0.1182 ± 0.0278	0.1310 ± 0.0254##	0.4019 ± 0.0099*∗∗*##	0.7932 ± 0.0635

Relative expression of RUNX2 protein	7	0.0163 ± 0.0017	0.0258 ± 0.0082##	0.0968 ± 0.0057*∗∗*	0.0762 ± 0.0158
	14	0.0210 ± 0.0026	0.0294 ± 0.0073##	0.1357 ± 0.0246*∗∗*	0.1401 ± 0.0260
	21	0.0175 ± 0.0046	0.0292 ± 0.0064##	0.1930 ± 0.0832*∗*	0.1500 ± 0.0386

Relative expression of Dlx-5 mRNA	7	0.9742 ± 0.0921	1.0566 ± 0.0504##	2.0851 ± 0.2293*∗∗*	2.1265 ± 0.4104
	14	1.0388 ± 0.0515	0.9120 ± 0.3276##	2.4911 ± 0.2984*∗∗*##	3.6416 ± 0.7365
	21	0.9527 ± 0.0644	0.8845 ± 0.0413##	3.3096 ± 0.3557*∗∗*#	4.5190 ± 1.3563

Relative expression of Dlx-5 protein	7	0.0216 ± 0.0114	0.0348 ± 0.0012##	0.1017 ± 0.0254*∗∗*	0.1161 ± 0.0230
	14	0.0253 ± 0.0064	0.0218 ± 0.0036##	0.0865 ± 0.0239*∗*#	0.1506 ± 0.0289
	21	0.0291 ± 0.0029	0.0447 ± 0.0109##	0.2042 ± 0.0234*∗∗*##	0.4216 ± 0.0362

Relative expression of osterix mRNA	7	0.8809 ± 0.1061	0.7559 ± 0.0399#	1.2075 ± 0.1732*∗∗*	1.3938 ± 0.4592
	14	1.0333 ± 0.0924	0.8624 ± 0.2732##	1.7024 ± 0.2401*∗∗*#	2.1167 ± 0.4100
	21	0.8365 ± 0.4278	0.6243 ± 0.0860##	2.3860 ± 0.2616*∗∗*##	6.9909 ± 1.6734

Relative expression of osterix protein	7	0.0171 ± 0.0024	0.0153 ± 0.0045##	0.0400 ± 0.0048*∗∗*#	0.0814 ± 0.0182
	14	0.0166 ± 0.0042	0.0209 ± 0.0028##	0.0947 ± 0.0147*∗∗*##	0.5140 ± 0.0287
	21	0.0260 ± 0.0057	0.0243 ± 0.0036##	0.2001 ± 0.0269*∗∗*##	0.4832 ± 0.0376

Values are mean ± sd.*∗*P < 0.05 and*∗∗*P < 0.01 compared with control group. ^#^P < 0.05 and ^##^P < 0.01 compared with BMP-2/VEGF group.

## Data Availability

Data in the present article has been displayed as figures and tables above.
